# Using Greedy Random Adaptive Procedure to Solve the User Selection Problem in Mobile Crowdsourcing

**DOI:** 10.3390/s19143158

**Published:** 2019-07-18

**Authors:** Jian Yang, Xiaojuan Ban, Chunxiao Xing

**Affiliations:** 1 School of Computer and Communication Engineering, Beijing Key Laboratory of Knowledge Engineering for Materials Science, University of Science and Technology Beijing, Beijing 100083, China; 2 Research Institute of Information, Beijing National Research Center for Information Science and Technology, Department of Computer Science and Technology, Institute of Internet Industry, Tsinghua University, Beijing 100084, China

**Keywords:** mobile crowdsourcing, user selection, marginalism principle, GRASP-AR

## Abstract

With the rapid development of mobile networks and smart terminals, mobile crowdsourcing has aroused the interest of relevant scholars and industries. In this paper, we propose a new solution to the problem of user selection in mobile crowdsourcing system. The existing user selection schemes mainly include: (1) find a subset of users to maximize crowdsourcing quality under a given budget constraint; (2) find a subset of users to minimize cost while meeting minimum crowdsourcing quality requirement. However, these solutions have deficiencies in selecting users to maximize the quality of service of the task and minimize costs. Inspired by the marginalism principle in economics, we wish to select a new user only when the marginal gain of the newly joined user is higher than the cost of payment and the marginal cost associated with integration. We modeled the scheme as a marginalism problem of mobile crowdsourcing user selection (MCUS-marginalism). We rigorously prove the MCUS-marginalism problem to be NP-hard, and propose a greedy random adaptive procedure with annealing randomness (GRASP-AR) to achieve maximize the gain and minimize the cost of the task. The effectiveness and efficiency of our proposed approaches are clearly verified by a large scale of experimental evaluations on both real-world and synthetic data sets.

## 1. Introduction

With the development of mobile networks and smart terminals, mobile crowdsourcing [1,2] has gradually evolved into a novel distributed problem-solving paradigm, which uses popular mobile users to collect and process data beyond the past possible range and has become an important research hotspot. The proliferation of smartphones makes mobile crowdsensing applications possible. Newzoo’s “2018 Global Mobile Markets Report” [3] shows that the number of global smartphone users has exceeded 3.3 billion so far. As mobile data and hardware become cheaper in the next few years, the number of smartphone users is expected to reach 3.8 billion by 2021, which means there are a large number of potential users in mobile crowdsourcing applications. Furthermore, smartphones are equipped with a variety of powerful built-in sensors, such as GPS, camera, accelerometer, microphone, etc., which enable crowdsourcing users to easily collect basic data of various applications and send sensing data to crowdsourcing platforms.

**Figure 1:**
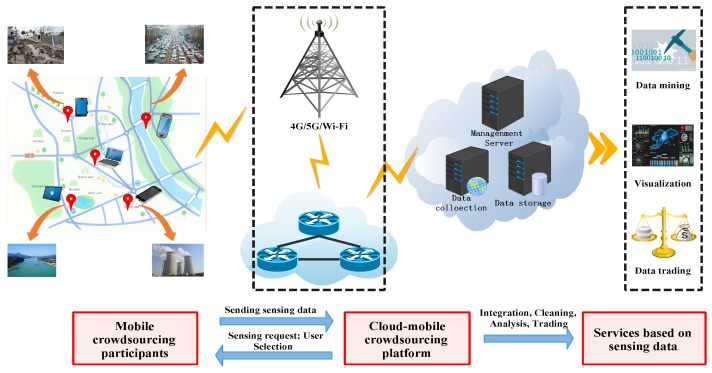
A mobile crowdsourcing system.

Inspired by the current popular smart city testbeds [4,5], a typical mobile crowdsourcing platform was proposed. As shown in Figure 1, the mobile crowdsourcing system consists of a large group of crowdsourcing participants distributed in a specific area and a crowdsourcing platform in the cloud, which is connected by mobile network or WiFi. Crowdsourcing participants(a.k.a, Users) use smart devices for road condition monitoring [6], pollution monitoring [7], location services [8], natural disaster assessment [9], etc., and send sensing data to crowdsourcing platforms. However, users consume their resources in performing tasks, such as batteries and computing power. Due to the diversity of individuals and smart devices, the cost of performing tasks is not the same. So they need to get different rewards from crowdsourcing platforms to continue their cooperation. Crowdsourcing platform provides a centralized management platform for sensing data receipt, integration, cleaning, analysis, and further building applications of interest, such as data mining, visualization and data trading, etc. In addition, the crowdsourcing platform has the right to publish tasks and select users.

To maximize crowdsourcing quality, the crowdsourcing platform needs to select the best subset of users available by matching the task requirements to the user’s profile, which is not easy. Crowdsourcing quality is affected by many factors. In addition to spatial location, existing research [10,11] has shown that user reputation, credibility, and time required to complete a perceived task have a significant impact on the quality of crowdsourcing. However, meeting the multi-objective constraints of mobile crowdsourcing remains a challenge. We need to consider not only the distance between the user and the task, but also the completion time of the task, the reputation and credibility of the users. In addition, under the above constraints, how to further select suitable users for the existing mobile crowdsourcing tasks to maximize the quality of crowdsourcing and minimize the incentive budget (i.e., minimum the total incentive cost of the selected users) is a major challenge for the crowdsourcing platform, which is called the mobile crowdsourcing user selection (MCUS) problem.

In truth, these two objectives are contradictory. If we improve one objective, the other will be reduced accordingly. As shown in literature [12], there is no optimal solution that can improve multi-objectives at the same time, but a set of pareto optimal solutions. To solve the problem, there are two standard approaches to formalize the problem: one is to find a subset of users that maximizes the crowdsourcing quality under a given budget; the other is to find a subset of users that minimizes the budget while meeting the minimum crowdsourcing quality requirements.

However, neither of the above methods is ideal in our context, because they all have an artificial predefined condition. Until the available users are selected, the mobile crowdsourcing platform often does not know how well the published tasks match the users. Therefore, it is unrealistic to require the crowdsourcing platform to predetermine a reasonable boundary to achieve a satisfactory compromise between multi-objective. The following two examples illustrate the limitations of both approaches.

**Example** **1.** *For simplicity, two potential users are shown in Table 1a, where Quality is the quality of service for users and Cost is the compensation for users. Assume that the upper limit of the budget cost of the crowdsourcing platform is pre-specified at $10. Not surprisingly, User X will be selected and not further inspect User Y. In fact, we can increase the cost slightly to get a more substantial profit. In other words, we increase the cost by $1, but we can get 80% of the service quality (improved by 30%). Arguably, it is worth spending some extra resources.*

**Example** **2.** *Similarly, two other users are shown in Table 1b. Assuming that the lower bound of the crowdsourcing quality is predetermined to be 80%, then User A will be selected and User B will not be further examined. In fact, achieving a cost reduction of $6 by slightly relaxing the requirements for crowdsourcing quality (i.e., 2%) is significantly cost effective.*

In practice, both of these scenarios are inevitable because we do not know the distribution of the different solutions and may miss a more desirable solution. Therefore, we need to find all the marginal points before we stop investigation. In other words, we will provide all the pareto-optimal solutions for the crowdsourcing platform to make a reasonable decision.

As mentioned earlier, we can’t use the previous methods to solve the user selection problem in mobile crowdsourcing, which is provably a NP-hard problem. To address this challenge, we have made the following significant contributions.

- We propose a scheme inspired by the principle of marginalism in microeconomics, and the problem of user selection in mobile crowdsourcing is formally defined in Section 3. Assuming that the same unit can be used to measure gain and cost in a crowdsourcing platform, we wish to stop selecting new users when the marginal gain is lower than the marginal cost. The marginal gain here refers to the difference between the benefit after and before the selection of users.
- In Section 4, we propose various gain-cost models driven by the Quality of Service (QoS), which provides a basis for evaluating the value of users’ contribution.
- We prove that user selection problem is NP-hard in Section 4.1. Then we propose a greedy random adaptive procedure with annealing randomness (GRASP-AR) to solve the user selection problem in Section 4.2.
- We conduct extensive experiments using real-world and synthetic data sets to evaluate our proposed algorithms on large-scale environment. The results show the effectiveness and efficiency of our proposed approaches in Section 5.

In addtion, we review the previous work on mobile crowdsourcing in Section 2. Section 6 concludes the paper and lay out a research agenda.

## 2. Related Work

In recent years, various researches on mobile crowdsourcing data management have focused on the following five core issues: task assignment [13,14], user selection [15], quality control [16], incentive mechanism [17,18,19], and privacy protection [20,21]. This article focuses on how to select a suitable group of users to accomplish a specific task. Therefore, the relevant research progress of the latter three issues is not covered in this section.

### 2.1. Task Assignment

In [22], they consider scheduling different sensing tasks assigned to smartphones in order to minimize the sensing energy consumption while ensuring the Quality of Sensing (QoSS). They proposed an integer linear programming (ILP) formula and two effective polynomial time heuristic algorithms for the corresponding minimum energy multi-sensor task scheduling (MEMS) problem, which has been well evaluated by extensive experimental evaluation. Zhao et al. [23] studied a target-aware task assignment problem that determines the optimal strategy for assigning each task to the right user to maximize the total number of completed tasks, and all users can reach their destination before the deadline after completing the task. To solve the problem, they used tree decomposition technology to separate the users into independent clusters and developed an efficient depth-first search algorithm. To et al. [24] also studied the issue of maximum task assignment. They use the spatial attributes of the problem, namely spatial distribution and user travel costs, to propose the minimum location entropy priority and close distance priority strategies to address these challenges. Wu et al. [25] proposed a real-time, budget-aware spatial crowdsourcing task allocation (RB-TPSC) model, with the goal of increasing task allocation rates and maximizing the expected outcome quality of the staff under a limited budget. The proposed RB-TPSC model can automatically make decisions on task allocation. In addition, Miao et al. [10] proposed a budget-aware task allocation method for spatial crowdsourcing, which is designed to help crowdsourcing platforms make task assignment decisions. Hassan et al. [26] focus on the problem of dynamic task assignment, a distance reliability ratio (DRR) algorithm based on combined fractional programming is proposed to maximize the reliability of the task and minimize the travel cost. DRR maximizes reliability and reduces travel costs by 80% compared to existing algorithms.

### 2.2. User Selection

Chen et al. [27] focus on the problem of how to choose suitable users to monitor environment by a crowdsensing network, while the total rewards for all selected users is not larger than the limited budget. To solve the problem, they first divide a big critical region into smaller regions of different size, and select some sampling points in the smaller region. Then, they designed a greedy algorithm to select users to cover the maximum sampling points while the total reward does not exceed the limited budget. In addition to time and space factors, Wang et al. [28] also included the data attributes into the mobile crowdsourcing, proposed a method of using the data attributes in the mobile crowdsourcing to select users, and then use the greedy algorithm to select the right users group. Finally, the effectiveness of the proposed method is proved by a large number of experiments on real data. He et al. [29] proposed a new user selection scheme, and designed a genetic algorithm based on greedy approximation and prediction trajectory to solve the problem that the existing user selection strategy does not perform well in vehicle-based group perception. For the multi-user multi-task assignment problem, Abououf et al. [11] proposed a group-based multi-task user selection model, which aim to assign multi-task to users, while maximizing the QoS of tasks and minimizing their completion time. Then the genetic algorithm and the tabu search algorithm were used to complete the user selection problem.

The focus of the above work is to optimize some of the performance for the user by reasonably assigning tasks. Some of them try to match a task to a suitable set of users (task assignment), while others attempt to prioritize users by considering their contributions (user selection). While most studies presuppose artificial constraints (e.g., budget constraint). As we described in the introduction, mobile crowdsourcing platforms are often unclear about the degree of match between published tasks and users. It is unrealistic to require crowdsourcing platform to predetermine a reasonable boundary to achieve a satisfactory compromise between multiobjective. Therefore, we need to redefine the issue of user selection.

## 3. User Selection Driven by the Gain-Cost Models

### 3.1. Problem Definition

In this section, we will formalize the definition of MCUS problem. For the readers’ convenience, the main notations used in this article are listed in Table 2.

**Definition** **1.** *(Multi-objective crowdsourcing task.) Let $T=\{T_{1},...,T_{i},...,T_{m}\}$, which represents m sets of multi-objective tasks, and each task is defined as a tuple form. $T=<L^{T_{i}},R^{T_{i}},Q^{T_{i}},RC^{T_{i}}>$, where $L^{T_{i}}$ is the current location of the task, which can be determined by latitude and longitude. $R^{T_{i}}$ is the minimum reputation requirement of the task, $Q^{T_{i}}$ is the minimum quality requirement of the task, and $RC^{T_{i}}$ is the range constraint of the task.*

**Definition** **2.** *(Crowdsourcing participant.) Crowdsourcing participants represent users involved in crowdsourcing task. Let $U=\{U_{1},...,U_{i},...,U_{n}\}$, which represents a set of n potential users, and each user is defined as a tuple form. $U_{i}=<L^{U_{i}},R^{U_{i}},D^{U_{i}},C^{U_{i}}>$, where $L^{U_{i}}$ represents the current location of the user, which can also be determined by latitude and longitude, and $R^{U_{i}}$ is the user’s reputation score. $D^{U_{i}}$ is the maximum traveling distance, which is determined by the user. $C^{U_{i}}$ represents the cost of the user to complete the task, and is also the compensation paid by the platform to the user.*

Reputation score can be obtained through the historical data of user in the crowdsourcing task. A high reputation means that QoS of users is higher. For example, it provides more correct results, takes less time to complete tasks, and so on. In addition, high quality of service means that users need to be paid more, which is in line with our intuition. The detailed calculations will be shown in Section 4.

**Definition** **3.** *(Gain for each task.) Gain is determined by the quality of service(QoS) provided by the user, which is denoted by G(Q). The gain satisfies the principle of diminishing marginal benefit [30] in microeconomics (i.e., increasing resources will gradually reduce the unit rate of benefit).*

**Definition** **4.** *(Cost of each task.) To obtain the service provided by the user for the task $T_{i}$, the crowdsourcing platform needs to pay for the selected users, which is also the cost that the crowdsourcing platform must pay for the service. The cost depends on the QoS of each user, so the cost can be expressed by $Cost=\sum_{i}^{\left|u\right|}C\left(U_{i}\left(Q\right)\right)$, where |u| is the number of users selected.*

Ideally, crowdsourcing platforms wish to maximize gain while minimizing cost. However, achieving both goals is often impossible. The traditional approach is to set constraints on one goal while optimizing on the other. Therefore, we can define the following two optimization problems with constraints.

**Definition** **5.** *(The $MCUS\_C_{1}$ problem.) Let U be a set of users, and $\delta_{c}$ be a budget on cost. The $MMCUS\_C_{1}$ problem finds a subset $\bar{U}\subseteq U$ that maximizes G($\bar{U}$) under constraint $C\left(\bar{U}\right)\leq \delta_{c}$.*

**Definition** **6.** *(The $MCUS\_C_{2}$ problem.) Let U be a set of users, and $\delta_{g}$ be a minimal requirement of gain. The $MMCUS\_C_{2}$ problem finds a subset $\bar{U}\subseteq U$ that minimizes C($\bar{U}$) under constraint $G\left(\bar{U}\right)\geq \delta_{g}$.*

As shown in Examples 1 and 2, neither of these constrained optimization goals is ideal. Inspired by the principle of marginalism [31] in microeconomics, we wish to stop selecting new users when the marginal gain is lower than the marginal cost, which is in line with the economic interests of the crowdsourcing platform. Therefore, the crowdsourcing platform needs to find a set of users with the largest profit (i.e., gain-cost). Assuming that both gain and cost can be measured in the same unit, such as dollars. Additionally, crowdsourcing platform can also apply a budget constraint, but unlike $MMCUS\_C_{1}$, balancing gain and cost don’t require budget constraint. Therefore, we define another goal that user selection.

**Definition** **7.** *(The MCUS Marginalism problem.) Let U be a set of users, $U=\{u_{1},u_{2},...,u_{n}\}$, and $\delta_{c}$ be a budget on cost. The MCUS Marginalism problem finds a subset $\bar{U}\subseteq U$ that maximizes $G\left(\bar{U}\right)-C\left(\bar{U}\right)$ under constraint $C\left(\bar{U}\right)\leq \delta_{c}$, which can be described as follows:*

(1) $$\begin{array}{c} Maximize\;G\left(\bar{U}\right)-C\left(\bar{U}\right)\\ s.t.=\left\{\begin{array}{c} C\left(\bar{U}\right)=\sum_{i=1}^{|\bar{u}|}c_{i}=\sum_{i=1}^{n}c_{i}x_{i}\leq \delta_{c}\\ x_{i}=0\;or\;1,i=1,2,\ldots ,n\\ |\bar{u}|\leq n. \end{array}\right. \end{array}$$

*where n is the total number of users in a mobile crowdsourcing system, $x_{i}$ denotes a binary variable: set to 1 if a user is selected and 0 if not. The constraint condition is optional, because we can provide the pareto optimal solution that meets the goal for the platform to make reasonable decisions according to its own constraints.*

### 3.2. Quality of Service (QoS) for Users

In mobile crowdsourcing, the quality of service (QoS) provided by users directly determines whether tasks can be completed. Relevant literature [10,11] show that users’ reputation and willingness to participate(WTP) contribute to the evaluation of QoS. When any one of these factors is reduced, the QoS of users will also be reduced, and vice versa. For instance, the higher the reputation, the more favorable the user received in the previous task, the higher the QoS, so the higher the probability of completing the current task. In addition, the quality of crowdsourcing is also determined by the WTP [32] of the user. The higher the WTP, the more human resources the crowdsourcing platform can obtain, which expands the range of users selected and thus increases access to first-rate services. However, the lower the WTP of users, the opposite. Therefore, we consider the reputation(the situation before the task was completed) and the willingness to participate of the users and model the product of these two factors as the standard for QoS in Equation (2).

(2) $$QoS^{U_{i}}=R^{U_{i}}\times WTP^{U_{i}}\in \left(0,1\right)$$

where *R* is the reputation of the users. Various models [33,34] have been proposed to evaluate the reputation. For simplicity, the popular beta reputation system (RBS) [35] is adopted. However, due to the limitations of RBS, the model does not work well when there are malicious users. For example, savvy users always try any solution that offers more benefits, and they may deliberately report unconfirmed information to obtain unreasonable rewards. And their reputation has not been significantly affected by once or twice unreasonable reporting. Without a penalty for dishonest reporting, crowdsourcing platforms will not only lose access to high-quality information, but also suffer serious financial losses. To avoid this situation, we have improved the RBS and calculated as follow:

(3) $$R=\frac{T+1}{T+F+2}*\xi \;\left(0\leq R\leq 1,\;T>0,\;F>0\right)$$

where *T* and *F* are the historical numbers of “correct” and “incorrect” results obtained by users when completing the crowdsourcing task. In addition, ξ is a weight associated with malicious events, which was calculated as follows:

(4) $$\left\{\begin{array}{c} if\;\;0\leq k<K\;\;then\;\;\xi =\lambda^{k}\\ if\;\;k\geq K\;\;then\;\;\xi =0 \end{array}\right.$$

where *k* is the malicious event of some user in the crowdsourcing task. *K* is the threshold for malicious events.

In addition to reputation, the QoS will also be affected by the willingness to participate of users. For example, user *A* who is far from the task location should have a lower willingness to participate than user *B* who is closer to the task location, because the distance will increase the cost of usrs. To reduce costs, crowdsourcing platform is also not worth recruiting users from far away. Based on the above analysis, we model the user’s willingness to participate and traveling distance as the following equation:

(5) $$WTP=1-max[0,min\left[log_{r}\left(D\left(L^{U_{i}},L^{T_{i}}\right)\right),1\right]]$$

where $D(L^{U_{i}},L^{T_{i}})$ is calculated as the Euclidean distance [36] between the user coordinates and the task coordinates. *r* is the range constraint of the task. As described in the literature [10], the crowdsourcing task is usually a micro-task, and users are not willing to participate in tasks that are too far away from them. Therefore, the crowdsourcing platform will provide the maximum range constraint of the task. Thus, WTP is a value between 0 and 1. Within the maximum range constraint of the task, if the user is closer to the task location, then WTP is closer to 1. If the location of the user is beyond the maximum range constraint of the task, then WTP = 0.

Based on the above analysis, substitute ((3)–(5)) into (2), then we can evaluate the $QoS^{U_{i}}$ of each user, and a task is completed by the cooperation of multiple users. We can increase the number of users until the marginal cost exceeds the marginal gain for the next incremental user. In other words, MCUS Marginalism problem aims to maximize gain of each task and ensure that a group of users is selected if adding a new user will increase the probability of the task success. This probability is defined as the number of correct reports submitted.

After the calculation of $QoS^{U_{i}}$, the probability of at least one success of the task is calculated as the binomial distribution of the total QoS obtained by the task. The $QoS^{total}$ of the selected a group of users is estimated as follow:

(6) $$QoS^{total}=1-\prod_{i=1}^{\left|u\right|}\left(1-QoS^{U_{i}}\right)$$

### 3.3. QoS-Based Gain-Cost Models

In this paper, we consider the effect of different gain-cost models [37,38] on user selection, take the QoS of user in Section 3.2 as an important gain factor, and establish three gain models.

- LinearGain assumes that the gain grows linearly with the QoS of users (For writing convenience, record it as Q(u)) and set G(u) = 100Q(u)
- QuadGain assumes that the gain increases quadratically with Q(u) and set $G\left(u\right)=100Q^{2}\left(u\right)$
- StepGain assumes that reaching a milestone of Q(u) will significantly increase gain and set:
  $$G\left(u\right)=\left\{\begin{array}{cc} 100Q(u) & :0\leq Q(u)<0.2\\ 100+100(Q(u)-0.2) & :0.2\leq Q(u)<0.5\\ 150+100(Q(u)-0.5) & :0.5\leq Q(u)<0.8\\ 200+100(Q(u)-0.8) & :0.8\leq Q(u)<1 \end{array}\right.$$

We assign the cost of a user in [10, 40] in four ways:

- RandomCost assigns a random integer cost in [10, 40];
- LinearCost assumes that the cost grows linearly with Q(u) and set C(u)=30Q(u)+10;
- QuadCost assumes that the cost increases quadratically with Q(u) and set $C\left(u\right)=30Q^{2}\left(u\right)+10$
- StepCost assumes that reaching some milestone of Q(u) will significantly increase cost and set:
  $$C\left(u\right)=\left\{\begin{array}{cc} 10 & :0\leq Q(u)<0.2\\ 20 & :0.2\leq Q(u)<0.5\\ 30 & :0.5\leq Q(u)<0.8\\ 40 & :0.8\leq Q(u)\leq 1 \end{array}\right.$$

## 4. Optimization Algorithm

In this section, we first analyze the complexity of solving MCUS Marginalism problem, and we rigorously prove that MCUS Marginalism problem is a NP-hard problem. Then we propose a greedy random adaptive procedure with annealing randomness(GRASP-AR), which attempt to overcome the limitations of deterministic algorithms (based on adding what is apparently the best element to the partial solution), that is, our algorithms strive to ensure that the solution is globally optimal because they explore the search space in a comprehensive way.

### 4.1. Complexity Analysis of MCUS Marginalism Problem

It is very important to solve the MCUS Marginalism problem with an efficient algorithm. Unfortunately, as we will prove next, MCUS Marginalism problem is a NP-hard.

**Theorem** **1.** *MCUS Marginalism problem defined in Defintion 7 is a NP-hard Problem.*

**Proof.**  The MCUS Marginalism problem can be proved by reducing $MCUS\_C_{1}$ problem. Further, we can prove that the decision version of $MCUS\_C_{1}$ is NP-complete. Next, we should find a known NPC problem and then try to reduce it.

We use the knapsack problem as a known NP complete problem. The knapsack problem is defined as follows. For an instance A of knapsack problem and a set of object $K=\{k_{1},k_{2},...,k_{n}\}$ with value and weight, where $k_{i}$ is represented by $v_{i}$ and $w_{i}$ respectively. The question is whether exists a set $\bar{K}\subseteq K$ that maximizes the value of members from $\bar{K}$ (i.e., $v_{max}\left(\bar{K}\right)$), and further $\sum_{i=1}^{|\bar{K}|}w_{i}\leq \eta$, where η is upper bound of the capability.

Next, we change instance *A* to an instance of $MCUS\_C_{1}$. We construct a user instance *B* with cost upper bound δ, and represent it as $U=\{u_{1},u_{2},...,u_{n}\}$. For each $u_{i}$, its gain and cost are $g_{i}$ and $c_{i}$, respectively. The $MCUS\_C_{1}$ problem is to find a set $\bar{U}\subseteq U$ to maximize the gain from the members of $\bar{U}$ (i.e., $G_{max}\left(\bar{U}\right)$), and further $\sum_{i=1}^{|\bar{U}|}c_{i}\leq \delta$. We assume that $\bar{K}$ can be used as a solution for instance *A*. By trying to select $|\bar{U}|$ users in the set *U*, the formed $\bar{U}$ can be used as a solution for $MMCUS\_C_{1}$.

With the construction approach of the solution, $\sum_{i=1}^{|\bar{U}|}c_{i}\leq \delta$ and a maximal $g_{max}\left(\bar{U}\right)$ imply $\sum_{i=1}^{|\bar{K}|}u_{i}\leq \eta$ and a maximal $v_{max}\left(\bar{K}\right)$, respectively.

Then, we can simply see that the reduction from A to B ends in polynomial time, since the knapsack problem is a NP-hard problem, so the $MCUS\_C_{1}$ is NP-hard. While MCUS Marginalism problem can be reduced to $MCUS\_C_{1}$, so MCUS Marginalism problem is also a NP-hard problem. □

### 4.2. GRASP with Annealing Randomness (GRASP-AR)

In the previous section, we have shown that MCUS marginalism problem is a NP-hard problem. As the scale of users continues to expand, solving MCUS Marginalism problem is limited by both memory and time. In practice, the number of decision variables increases exponentially as the scale of users increases. Even if computing resources can increase indefinitely, the exact solution may not be found in a reasonable amount of time. To address the problem, we proposed a greedy randomized adaptive search procedure with annealing randomness as a trade-off between computation time and quality of found solutions.

GRASP [39,40] is a multi-start meta-heuristic algorithm, which consists of two phases: construction phase and local search phase. In the construction phase, the iterative constructs a feasible solution, one element at a time. The greedy randomized algorithm is first used to select the top-*k* candidates from the generated profits, and the best solution is selected from multiple iterations. The algorithm is adaptive and provides a good initial solution while maintaining a certain degree of diversification to avoid convergence toward local optima. In the local search phase, we introduce a simulated annealing (SA) [41] meta-heuristic, whose effectiveness depends on the fact that it accepts a non-improved solution within a certain probability. In other words, it can accept a solution that is worse than the best solution found at the time. The probability of this solution depends on the degree to which the new solution differs from the optimal solution and a further parameter, the synthesis temperature excited by the metallurgical annealing process. As the value of the parameter decreases gradually, the randomness of the method also gradually decreases (the lower the value, the lower the randomness). Because SA can search for feasible solutions in a larger range, making it possible for the algorithm to jump out of the local optimal solution and find a global optimal solution.

**Algorithm 1:** GRASP-AR(U,λ,k).

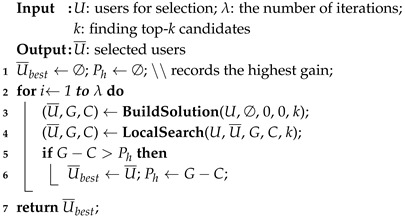



Algorithm 1 introduces the main processing phases of GRASP-AR. The algorithm ensures that a subset of users with the largest marginal gain is found. The algorithm performs λ iterations. In each iteration, the construction phase constructs an initial solution $\bar{U}$, then the local search phase uses a simulated annealing strategy to further find a viable solution in the $\bar{U}$ neighborhood. Finally, it returns the best solution from all iterations.

The construction process of greedy randomized is given in Algorithm 2. First, a given set of candidate users is initialized and a group of users is added iteratively in a greedy randomized. In each iteration (Step. 2–14), we select the user with the maximum incremental gain from the remaining user set *U*∖$\bar{U}$ (Step. 5). In other words, we check one by one whether the maximum gain achieved by the remaining users can exceed the current best solution, and skips the user if not. Next, Step. 6 evaluates the difference between the marginal gain and marginal cost of the selected users. The top-*k* user sets are selected in this way (Step. 7–10). Finally, Step. 12–15 choose subset of users with the highest gain.

**Algorithm 2:** Procedure BuildSolution($U,\bar{U},G,C,k$).

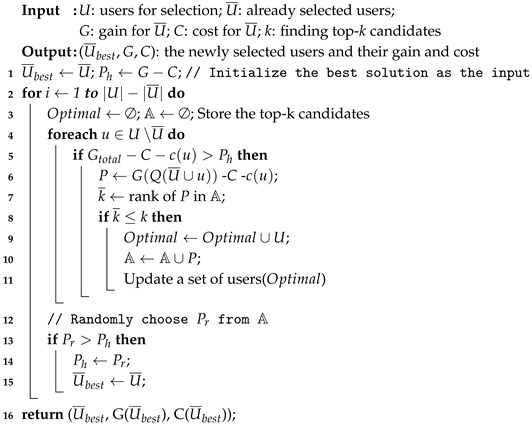



**Algorithm 3:** Procedure LocalSearch(U, $\bar{U}$, G, C, k).

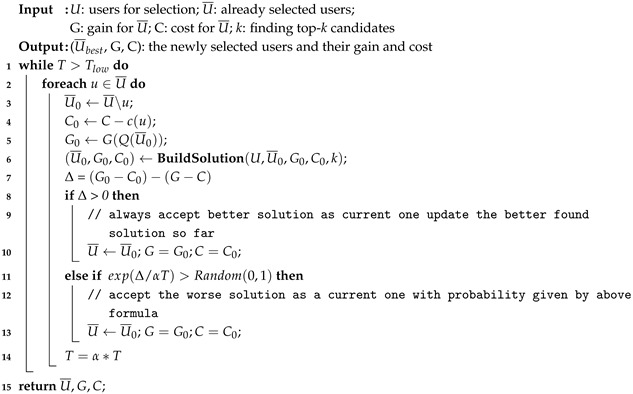



The local search phase takes the initial solution as input and iteratively explores its neighborhood for a better solution. In this work, we introduce SA meta heuristic method. SA is a general probability algorithm. Its starting point is based on the annealing process of solid materials in metallurgical processes. It is initialized with a parameter called temperature, denoted *T*, which accepts a non-improved solution within a certain probability. According to the cooling rate factor α, the temperature slowly drops during execution. The lower the temperature, the lower the probability of choosing a worse solution in the next iteration.

The proposed SA is given in Algorithm 3. First, it takes the construction phase of the solution as input, which is the best solution found so far. Next, in each iteration, it compares the current solution with (1) the solution of removing *u* (2) the candidate solution with the remaining user subset replacement *u*, and represents the difference between them as Δ. If the candidate solution is better, consider it as the current solution and update the best solution found. If the candidate solution is worse, it is accepted as the new current solution with the probability exp(Δ/αT). Finally, the temperature decreases with the cooling factor until the preset minimum temperature stops.

## 5. Experimental Evaluation

The purpose of this section is to examine the performance of our algorithm using both real-world and synthetic datasets. We compare GRASP-AR with three baseline approaches on a series of mobile crowdsourcing tasks. First, we describe our experimental design, then we analyze the results under various experimental settings.

All experiments were coded in Python under Windows 10 for Education platform on an Intel Core i7 2.8 GHz processor with 16 GB of RAM.

### 5.1. Experiment Design

#### 5.1.1. Datasets

**Figure 2:**
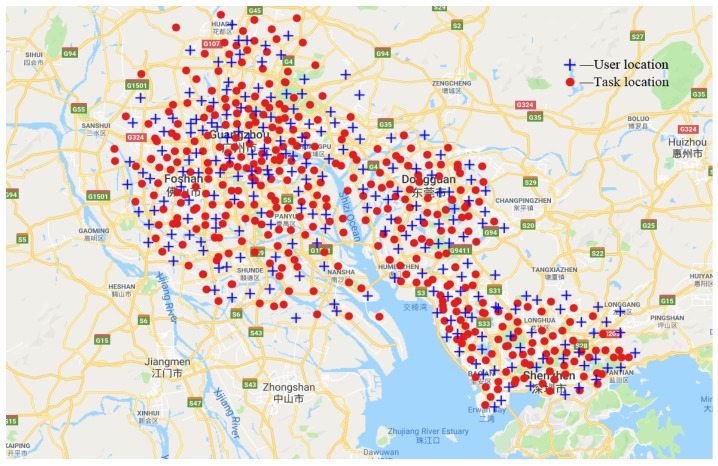
A real-world data set of task allocation.

**Real-world data**: To evaluate the performance of the GRASP-AR algorithm, we used a task assignment data set [42] published by the China Society for Industrial and Applied Mathematics, which contains 835 tasks and 1877 users from four Chinese cities (i.e., Guangzhou, Foshan, Shenzhen and Dongguan). The distribution of task locations and user locations are shown in Figure 2. In the experiment, we set the parameter $RC^{T_{i}}$ = 50 km. In addition, the dataset also provides the reputation of all users, which is scaled to a range of (0, 1). The dataset parameter settings are summarized in Table 3.

**Synthetic data**: We follow the existing methods [43,44] to generate synthetic data. Assuming that the location of tasks and users are distributed in a 1 km × 1 km 2D space, then longitude and latitude are uniformly distributed in (0, 1). In addition, each user has a reputation score *R* that reflects the completion of tasks in the past. For simplicity, the reputation of users is randomly selected from the range of (0, 1). The *n* users and *m* tasks are selected from {100, 200, 300, 400, 500, 600, 700, 800, 900} and {100, 200, 300, 400, 500, 600, 800} respectively. We use Euclidean distance to quantify the traveling distance between tasks and users. The synthetic data parameter settings are summarized in Table 4, where default values are highlighted in bold.

#### 5.1.2. Baseline Algorithms

To evaluate our GRASP-AR algorithm, we introduce three other algorithms. One is Particle Swam Optimization (PSO) algorithm as in [45]. PSO is an optimization method based on Swarm Intelligence, which originated from the research on predation behavior of birds. Compared with other modern optimization methods, PSO is featured by few parameters to be adjusted, simple operation and fast convergence, which has become a hotspot in the field of modern optimization methods. We have set the size of the swarm to 30, the inertia weight linearly varied between 0.9 to 0.4, and the acceleration coefficients to 2. Also, we compare our GRASP-AR algorithm with its two simplified versions, namely Greedy Randomized Algorithm (GRA) and Simulated Annealing (SA). The former one invokes *BuildSolution* with *k* = 1 (see Algorithm 2). The latter one essentially invokes *LocalSolution* (see Algorithm 3). The difference is that the initial solution for SA is generated randomly.

Additionally, we adjust parameters separately and incrementally, i.e., when a parameter is tuned, others are set to the default values. While next parameters are investigated, previous ones are taking the best value found so far. Table 5 lists the range of parameter values and default value for GRASP-AR algorithm.

### 5.2. Evaluation Results

#### 5.2.1. Comparison of Three Selection Schemes under Different Gain-Cost Models Using Real Dataset

We considered three user selection schemes mentioned in Section 3.1: (1) $MCUS\_C_{1}$ with $\delta_{c}$ = $\frac{G(1)}{2}$ (G(1) corresponds to the maximum gain), (2) $MCUS\_C_{2}$ with $\delta_{g}$ = G(0.8), and (3) MCUSMarginalism with $\delta_{c}$ = ∞. We use the default experimental parameters in Table 3 for each scheme.

**Figure 3:**
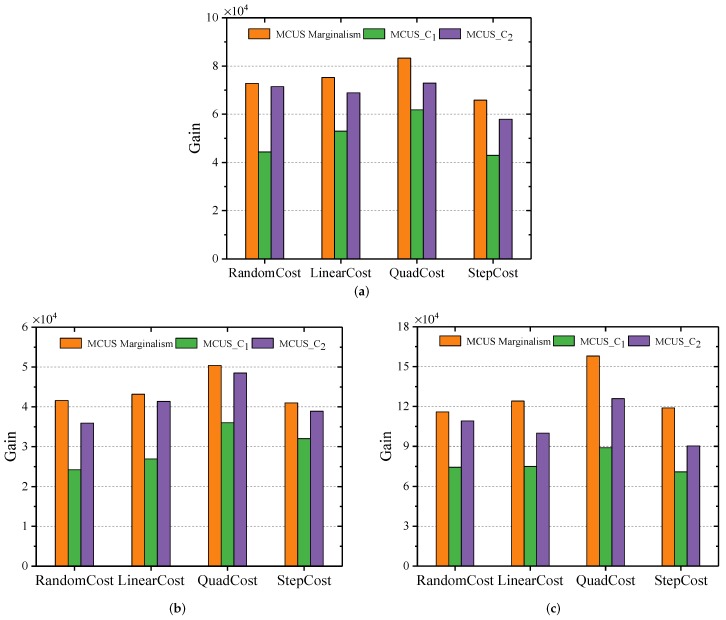
Comparison of three selection schemes. (**a**) LinearGain; (**b**) QuadGain; (**c**) StepGain.

To compare the effects of different gain-cost models on user selection, we used LinearGain, QuadGain, StepGain and various cost models on the real-world dataset. As shown in Figure 3, different gain models show the same patterns. Specifically, the user selection scheme (MCUSMarginalism) we proposed achieved the highest gain in most cases. Taking Figure 3a as an example to illustrate, MCUSMarginalism is nearly 32% higher than $MCUS\_C_{1}$ and 9.6% higher than $MCUS\_C_{2}$ in terms of average gain. This is because $MCUS\_C_{1}$ costs a lot, and $MCUS\_C_{2}$ always stops at a fairly low gain. This difference is more obvious in the StepGain model.

#### 5.2.2. Performance Comparison of Four Algorithms under Different Gain-Cost Models Using Real Dataset

We used real-world dataset and generated a total of six instances based on different gain-cost models. GRASP-AR, PSO, GRA, SA were used to obtain the best value, the worst value, the mean value, and the standard deviation (S.D) for solving each instance 20 times independently.

Table 6 shows the results of RandomCost and LinearCost. Firstly, in terms of the results of best value, we observed that GRASP-AR achieved 5 best results in 6 instances, SA achieved the best results in only one instance, while others did not. Secondly, the performance of various StepGain models is poor, which may be due to the discontinuity of the gain with the increase of QoS.

In addition, we used GAP to evaluate the statistical characteristics of the average performance of all algorithms. The calculation of GAP is the same as [38], which is measured by the relative difference between the best value and the average value:

(7) $$GAP=\frac{\left|best-mean\right|}{best}\times 100\%$$

**Figure 4:**
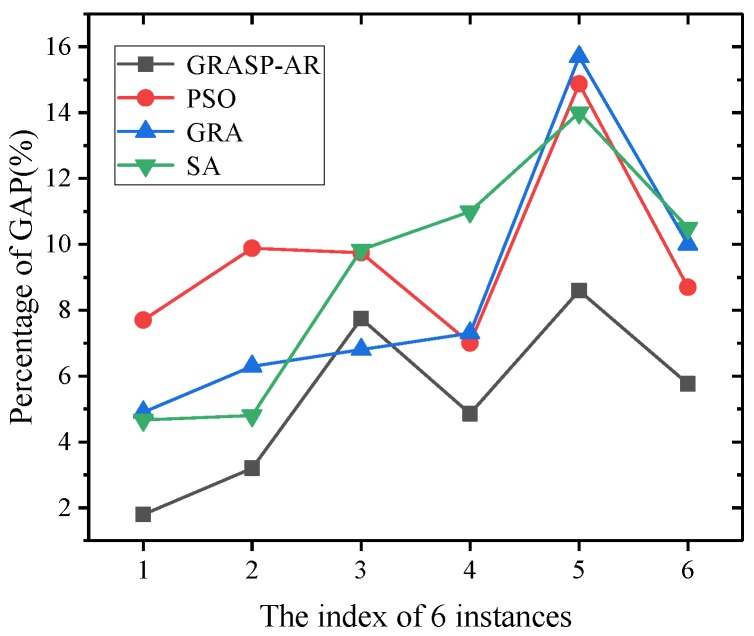
GAP fitting curve of real-world dataset under different gain-cost models.

Then, we can compare the average performance of all algorithms by GAP fitting curve. The closer the gap fitting curve is to the abscissa axis, the better the average performance of the algorithm. The fitting curves of each algorithm are given in Figure 4. It can be seen that the performance of GRASP-AR is the best among the four algorithms, because the gap fitting curve is the closest to the abscissa axis.

**Figure 5:**
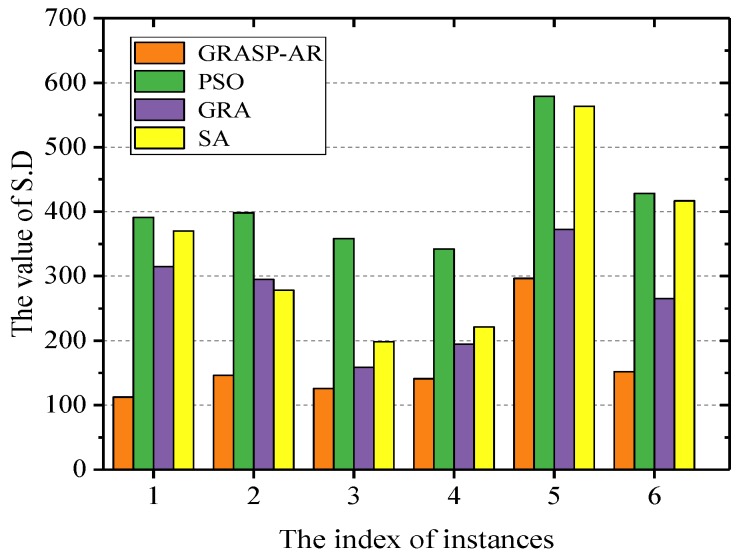
Standard deviation (S.D) histogram of real-world dataset under different gain-cost models.

Moreover, we plot the histogram according to S.D and evaluate the stability of the four algorithms by the distribution of the columns. As shown in Figure 5, the stability of GRASP-AR is much higher than the other three algorithms.

#### 5.2.3. Performance Comparison of Various Parameter Combinations for GRASP-AR Using Synthetic Dataset

**Figure 6:**
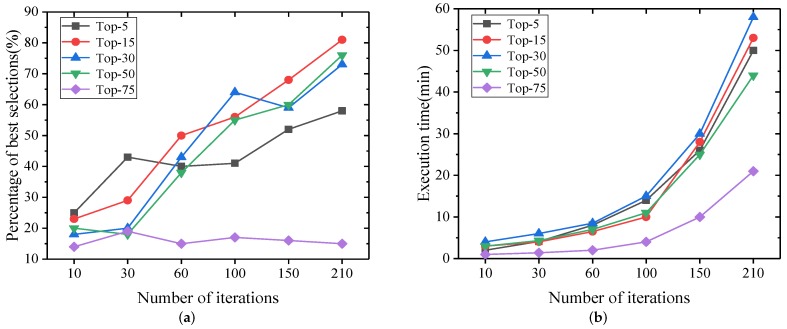
Effectiveness and efficiency of various parameter combinations for GRASP-AR. (**a**) Percentage of Best Selections; (**b**) Execution Time.

We used LinearGain and RandomCost to find the best selection percentage and running time for different combinations of λ and *k*. Figure 6 shows the results of our experiment. We have three conclusions. First, there is no doubt that the more iterations, the longer the time, but the more iterations, the better the results. Second, better results are often obtained when *k* = 15. This is because if the value of *k* is too low, the best solution may not be found, and if the value of *k* is too high, it is close to random search, which will reduce the quality of the results. Finally, when *k* increases gradually, the running time increases, while *k* exceeds 50, the running time decreases. This is because when *k* is large, it is less likely to find a better solution in the construction phase, so there are fewer iterations in the local search phase.

#### 5.2.4. Performance Comparison of Four Algorithms under Different Number of Tasks Using Synthetic Dataset

The experiment in this section aims to evaluate the performance of the algorithm according to the number and location of different tasks when determining the number and location of users. We consider 200 users and gradually increase the number of tasks from 100 to 1000. Moreover, we use LinearGain and RandomCost models.

**Figure 7:**
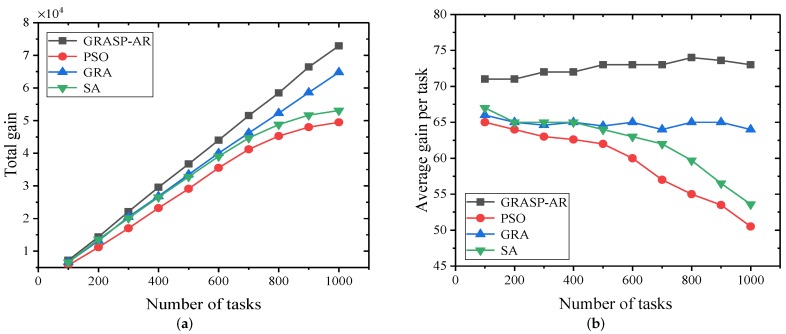
Effectiveness and efficiency of various parameter combinations for GRASP-AR. (**a**) The total gain achieved for all tasks vs. the number of tasks available. (**b**) The average gain achieved per task vs. the number of tasks available.

As shown in Figure 7a,b, while all algorithms aim to maximize the gain of the task, GRASP-AR achieves the maximum (total) gain of all tasks and the average gain of each task, respectively. Specifically, GRASP-AR performs approximately 32%, 17% and 14% better than PSO and GRA and SA, respectively. It is no surprise that GRASP-AR provides a good starting point and maintains a variety of solutions in the construction phase, which provides a good foundation for the final user selection. Secondly, the local search phase explores the neighborhood of the solution and further improves the solution. Furthermore, we can see that the SA cannot be extended over a large number of tasks, which significantly reduces the task gain.

#### 5.2.5. Performance Comparison of Four Algorithms under Different Number of Users Using Synthetic Dataset

The experiment in this section aims to evaluate the performance of various algorithms according to different number of users when determining the number and location of tasks. We considered 50 tasks and gradually increased the number of users from 100 to 800. Similarly, we use the linear gain and random cost models.

**Figure 8:**
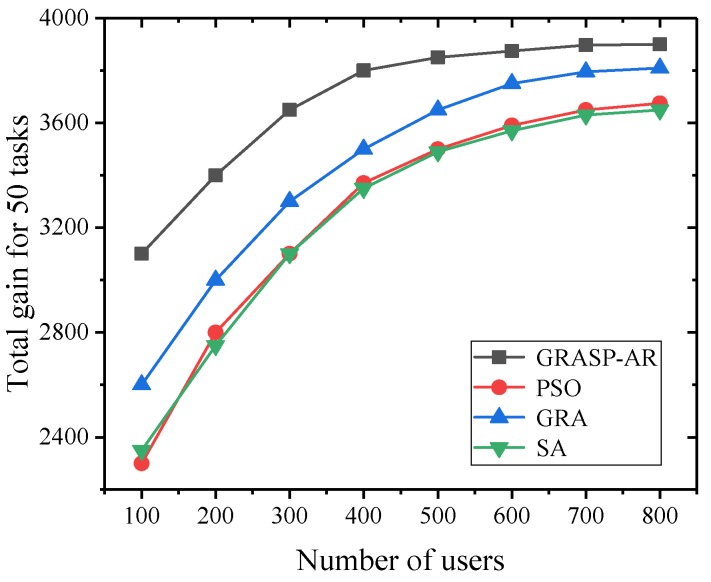
The total gain achieved for 50 tasks vs. the number of users available.

Figure 8 shows the effect of available users on the total gain of 50 tasks. It can be seen that with the increase of the number of potential users, the contribution of the four algorithms to the total gain of 50 tasks is increasing, and their fitting curves are getting closer. This is because the more users in the search space, the more potential users to provide high-quality service, and a better subset of users can be found for the task. It is worth noting that the performance of GRASP is still higher than other algorithms. It is 15.8%, 14.3% and 8.7% higher than SA, PSO and GRA, respectively, in terms of average gain.

## 6. Conclusions and Future Work

This paper studies the problem of user selection in mobile crowdsourcing. We propose a marginalism problem of user selection to achieve maximize the quality of service of task while minimizing the total incentive cost. In addition, in order to estimate the contribution of users to task, we construct various gain-cost models driven by willingness to participate and reputation of the users. To address user selection marginalism problem, we develop a greedy random adaptive procedure with annealing randomness that can efficiently retrieve the potential user selection solution feasible space. Experimental results show the effectiveness of our algorithms on both real-world and synthetic datasets.

There are many opportunities to extend this work for full-fledged user selection for moblie crowdsourcing. We next lay out a research agenda by describing several future research directions.

- User contribution measures: In addition to the user’s willingness to participate and reputation, there are a variety of indicators to evaluate the user’s contribution to the task, such as credibility, task completion time, etc. Future work includes efficiently estimating user contribution and selecting user given these new measures.
- Improved gain and cost models: When we have multi-dimensional user contribution measures, the gain model can be much more complex. Similarly, the cost model may be more complex based on some specific pricing strategies [46,47]. Future work includes designing more complex gain-cost models and studying their effect on user selection.

## Figures and Tables

**Table 1:** Two examples.

(a) Example 1			(b) Example 2		
User	Quality	Cost	User	Quality	Cost
X	50%	$10	A	80%	$11
Y	80%	$11	B	78%	$5

**Table 2:** Main notations used throughout the paper.

Notation	Description
$T_{i}$	A task published by crowdsourcing platform
$L^{T_{i}}$	The location of a task $T_{i}$
$R^{T_{i}}$	The minimum reputation requirement of task $T_{i}$
$Q^{T_{i}}$	The minimum quality requirement of task $T_{i}$
$RC^{T_{i}}$	The maximum range constraint of task $T_{i}$
$U_{i}$	A mobile crowdsourcing task participant
$L^{U_{i}}$	The location of user $U_{i}$
$R^{U_{i}}$	The reputation of user $U_{i}$
$D^{U_{i}}$	The maximum traveling distance constraint set by user $U_{i}$
$C^{U_{i}}$	The cost of user $U_{i}$ to complete the task
Gain	The profit per task
Cost	The total cost per task
WTP	The willingness to participate of the user
$QoS^{U_{i}}$	QoS provided by user $U_{i}$
$QoS^{total}$	QoS provided by a group of users

**Table 3:** The real-world data parameters and their values.

Parameter	Description	Value
*m*	Number of crowdsourcing tasks	835
*n*	Number of users	1877
$RC^{T_{i}}$	Range constraints for each task	50 km
*R*	Reputation of user	(0, 1)

**Table 4:** The synthetic data parameters and their values.

Parameter	Description	Range of Values
*n*	Number of users	{100, 200, **300**, 400, 500, 600, 700, 800, 900}
*R*	Range of the reputation of a user	[0, 1]
*m*	Number of crowdsourcing tasks	{100, 200, 300, **400**, 500, 600, 700, 800}
*g*	Grid size	1 km × 1 km squares

**Table 5:** The GRASP-AR algorithm parameters and their values.

Parameter	Description	Range of Values	Default Value
*k*	Top-k candidates	{5, 15, 30, 50, 75}	15
λ	Number of repetitions	{10, 30, 60, 100, 150, 210}	150
α	Cooling rete factor	{0.990, 0.995, 0.999, 0.9995, 0.9999}	0.995
*T*	Initial temperature	{200, 400, 600, 800, 1000}	400
$T_{low}$	Termination temperature	0.1	0.1

**Table 6:** Performance Comparison of GRASP-AR, PSO, GRA and SA.

Index	Gain	Cost	Algorithm	Best	Mean	Worst	S.D
1	Linear	Random	GRASP-AR	72,768	71,395	68,742	112.56
			PSO	69,128	63,746	55,221	437.24
			GRA	70,153	66,712	58.968	314.78
			SA	70,994	67,475	61,827	294.28
2		Linear	GRASP-AR	75,245	72,847	70,328	146.15
			PSO	71,824	64,723	58,863	398.16
			GRA	73,080	68,451	62,058	295.14
			SA	72,993	69,418	64,862	278.35
3	Quad	Random	GRASP-AR	41,582	38,358	35,210	125.85
			PSO	38,692	34,917	31,026	358.14
			GRA	39,822	37,132	34,328	158.86
			SA	40,217	36,258	33,461	198.53
4		Linear	GRASP-AR	43,158	41,062	38,894	140.85
			PSO	40,558	38,258	34,016	342.10
			GRA	41,139	38,153	35,927	194.85
			SA	41,056	36,534	33,635	221.52
5	Step	Random	GRASP-AR	115,816	105,848	97,451	296.43
			PSO	104,487	88,945	80,032	578.82
			GRA	109,922	92,628	85,396	372.52
			SA	116,132	99,863	84,150	606.21
6		Linear	GRASP-AR	124,136	116,980	109,628	152.18
			PSO	105,662	96,463	86,916	428.47
			GRA	115,132	103,558	90,284	265.37
			SA	113,472	100,824	88,153	416.85
